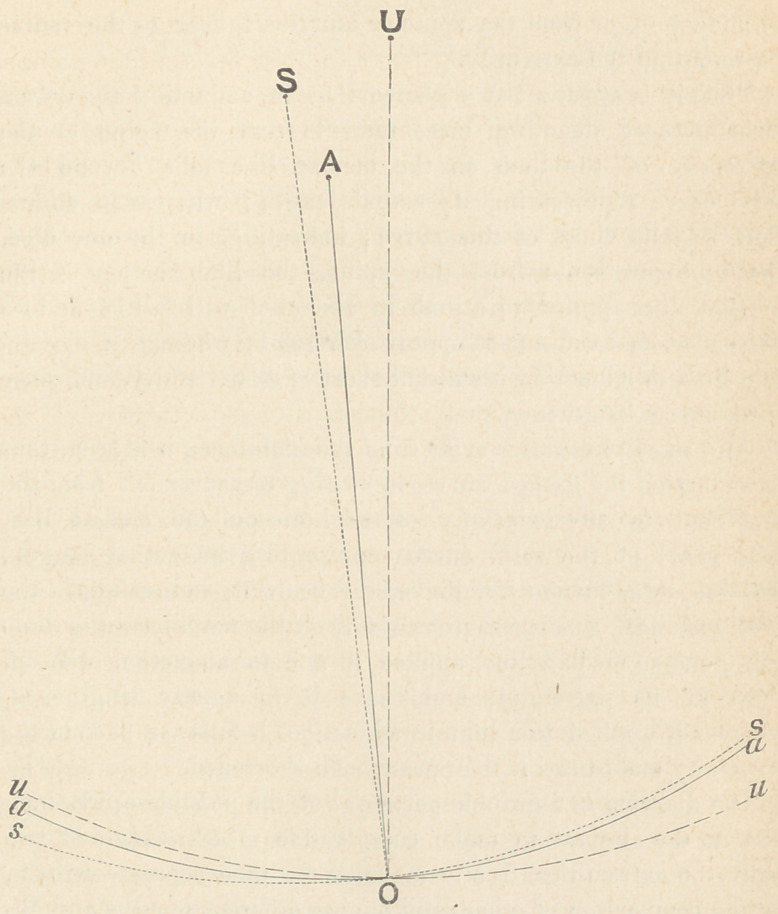# Measurements of Extremities

**Published:** 1878-05

**Authors:** John Bartlett


### A CONSIDERATION OF SOME OF THE ERRORS INCIDENT TO THE ORDINARY METHODS OF DETERMINING THE RELATIVE LENGTHS OF THE LOWER EXTREMITIES,

### With a Description of an Instrument Designed to Secure an Approximation to Precision in such Measurement.

By John Bartlett, M. D.

(*Summary of a paper read before the Chicago Medical Society, March* 4, 1878.)

The anterior superior spinous processes of the ilium, are ineligible as points of measurement, because practically, they are not determinable with precision.

The umbilicus is objectionable in that it may incline from the median line.

By the weight and pulley method of treatment of fracture of the thigh, the pelvis is inclined toward the injured limb. This inclination of the pelvis is equivalent to abduction of the thigh. To approximate the sound limb to the injured one, under such circumstances, is to adduct it.

As frequently dressed, the injured thigh is in a state of slight flexion.

A lower extremity in abduction, measured from the iliac spine, as contrasted with its fellow lying in true line with the pelvis (with the inner margin of the heel on the median line), has the appearance of being shortened in a measure, bearing a direct proportion to the degree of abduction. Thus, if the limb be abducted one foot (measuring the degree of abduction on a line drawn from the inner side of the heel, perpendicular to the median line), the apparent shortening will be three quarters of an inch, nearly. If it be abducted two feet, the apparent shortening will be one and a half or two inches.

Measured from the spine, a limb in adduction contrasted with its fellow measured when at the median line, presents one quarter of an inch of lengthening for six inches of adduction, and three-eighths of lengthening for one foot of adduction, approximately.

Measured from the umbilicus, a limb in abduction, as contrasted with its fellow lying at the median line, appears to be lengthened for one foot of abduction eleven-sixteenths of an inch ; for two feet of abduction nine-sixteenths of an inch, nearly.

A limb in adduction, measured from the umbilicus, as contrasted with its fellow at the median line, appears to shorten rapidly ; about one and a half inches for one foot of adduction.

A limb slightly flexed upon the pelvis, contrasted with its fellow lying in line with the body, whether measured from the umbilicus or spine, appears to be shortened in proportion to the degree of flexion, the amount being about one-eighth of an inch for one inch of elevation of the heel, two-eighths for two inches of elevation, and three-eighths for three inches.

Errors in measurement are apt to arise from the varying degree of rotation of the limb inward or outward, and when the measurement is taken from the sole of the foot, or by passing a loop about the foot, marked errors may arise from the varying degrees of extension of the foot at the moment of measurement.

If the sound limb, when used as a standard of measurement, does not lie in a line with the pelvis, but in adduction, a combination of errors arises, and the injured extremity will appear lengthened or shortened by an amount equal to the sum of the errors of abduction and adduction.

The instrument designed to eliminate these errors, consists of a pelvic arc of wood, intended to span over the pelvis, and to rest by its lower concave edge on the iliac spines. On its inferior face, this arc is marked from centre to extremities in inches, in order that it may be set as a base line on the spines, so that its centre shall be equi-distant from each, and so that a tangent drawn parallel to the ends of the arc, may be parallel to a line passing between the spines. Projecting perpendicularly from the lower surface of the arc, is a thigh piece, curved somewhat backward, that its inferior end may rest upon the bed, and connected to this by a knee-joint, is a leg piece extending some inches below the feet. This leg piece has moving up and down upon it, by means of a screw, a rule graduated in inches from the centre outwardly, and adjusted in exact parallelism to the supposed tangent of the arc, to which reference has been made. Upon this rule slide "riders," which, when a measurement is being taken, are brought directly beneath the point of intersection of two lines drawn upon the soles of the feet, the one transverse, directly under the ankle joint, where the movements of the foot in extension and flexion are least appreciable, the other marking the central line of the sole.

The portion of the instrument thus far described, may be termed the measuring piece, the part to be mentioned may be called the base.

For any purpose of accuracy, more definite, determinate points of measurement should be taken than those furnished by the iliac spines. As a more suitable bench mark, corresponding points upon the upper margins of the crests of the ilia are chosen, upon which to apply the base of the instrument. This, a kind of body yoke, consists of two rectangular wooden plates adapted to the sides of the abdomen, held in position by a girdle of iron rods, and thrust firmly down upon the iliac crests by tension of the thoracico-humeral muscles communicated by crutch-pieces, the extremities of which bear upon the upper margins of the side-plates. Jutting outwardly from the lower edges of these plates, are discs of metal to which the ends of the arc piece of the measuring portion are immovably clamped.

The instrument having been secured upon the pelvis by the means just detailed, the thigh and leg pieces take position between the limbs of the patient, with the rule resting below the feet. The degree of abduction of the injured limb being read upon the rule, the uninjured extremity is placed in a corresponding position not only of abduction, but of flexion and rotation. The limbs being thus symmetrically adjusted, the rule, by the slow windlass motion, is approximated to the soles of the feet, till the edge of one of the riders impinges against the point of intersection marked thereon. The distance at which the opposite rider stands from the measuring point on the sole of the foot of the shorter leg, will be the difference in the lengths of the limbs required.

In view of the objections to the use of points of measurement for the lower extremities apart from, and out of the axis of, the limb, it would be better to select one as nearly as possible over the centre of motion of the femur. Such a point may be determined on the sound side with a good degree of accuracy, in the manner to be described; and a corresponding point on the injured side may be located by comparative measurement. Flex the thigh upon the pelvis, and mark with India ink or nitrate of silver, a line corresponding to the fold of the groin, indicating the line of hinging of the thigh on the body. Then choose a point of provisional measurement, from which the limb will give the same measurement at convenient degrees of abduction and adduction. Such a point is found on a level with the iliac spines, and about midway between the "spine" and the median line. Adduct the limb some inches, and trace on the abdomen with India ink and pencil, the line which the tape makes from the provisional measuring point, a few inches downward. Then abduct the limb carefully, avoiding errors of rotation and flexion, the eye meanwhile resting on the tape measure, at the ankle or foot, as it indicates, first lengthening and then shortening. Abduction must cease exactly at the point where the tape gives the initial figure of measurement, that is, where the limb in abduction measures the same as at the beginning, when resting in adduction.

The course of the tape in this position must now be traced from the provisional centre of motion. The angle formed by this line, with the one just drawn, is bisected, and the point at which the bisecting line intersects the groin line, lies over the centre of motion of the hip joint. Measurements from it may be taken without regard to the relative positions of the limbs in abduction or adduction. Instead of bisecting the angle formed by the tape line as a radius moving about the point of provisional measurement, the chord of the arc described by the heel in the change from adduction to abduction may be bisected upon a thin board placed under the heel. While the limb rests on the bisecting line, the tape stretched between the provisional point and the point of original selection at the extremity of the limb, will intersect the groin line over the centre of the head of the femur.

A corresponding point in the opposite groin is determined by comparative measurements from the lower line of the groin, the pubis, iliac spines, and the umbilicus. The two points thus obtained may be utilized in this way: The two ends of a twine tied at its middle into the ring of a tape-line, are held by an assistant one upon either spine, the one or the other being lengthened or shortened, as may be necessary to enable the surgeon drawing upon the tape, to place its ring exactly over the centre of motion point. This being effected, the measurement of the sound limb is taken and noted down. The ring of the tape-line is then placed in position over the centre of motion on the injured side, and corresponding measurements are made.

The above plan of finding a point over the centre of motion, is tedious and troublesome, and is far less convenient, and perhaps not more correct than this simple procedure: trace a groin line as directed above, and intersect it by a line drawn parallel to, and on the outer side of the femoral artery, distant from its central line the mean width of the index finger of the patient. The point of intersection of this, with the groin line, for practical purposes, may be regarded as over the centre of the head of the femur.

The facts given in the preceding summary may be illustrated by the accompanying geometrical diagram. The points A, S, U, O, represent the relative positions, as taken from the skeleton, respectively, of the centre of motion of the hip joint, of the anterior superior spinous process of the ilium, of the umbilicus, and of the inner edge of the heel. The radii AO, SO, UO, represent, the former the limb, and the latter two, measurements as taken from the "spine" and the umbilicus, respectively. Any imaginary line joining the several centres with any point in the solid, dotted or dashed curves may stand for the position of the limb, or of the tape from the spine or from the umbilicus respectively.

It will thus be seen, by contrasting the three circumferences, what relation any points of measurement, whether in abduction or adduction, or from the spine or umbilicus, bear to the radius representing the extremity.

Thus it is apparent at a glance that in abduction the dotted circumference denoting measurement from the spine to the extremity of the limb on the median line falls beyond the solid curve representing its actual length; whereas in adduction it falls short of this curve; indicating, in the one case, that measurements from the spine, the limb being on the median line, applied to a limb in abduction will fall beyond its extremity, and cause it to appear shortened; whereas, if applied to a limb in adduction it will fall short of its extremity, and cause it to appear lengthened.

And so as to measurements from the umbilicus, it is seen, that in abduction the dashed curve, indicating measurement from the umbilicus to the extremity of the limb on the median line, falls short of the solid curve representing its actual length; whereas, in adduction it falls rapidly below it, indicating, in the first instance, that measurements from the umbilicus, the limb being on the median line, applied to one in abduction will fall short of its extremity, and cause it to appear lengthened, whereas if applied to a limb in adduction, it falls far beyond the extremity and causes it to appear much shortened.

The diagram is a graphic portrayal of the possible errors incident to the attempt to make comparative measurements of two radii, the extremities, free to move about their separate centres, by the comparison of other radii having different lengths and different centres of motion.

## Figures and Tables

**Figure f1:**